# Comparative study between intrathecal dexmedetomidine and intrathecal magnesium sulfate for the prevention of post-spinal anaesthesia shivering in uroscopic surgery; (RCT)

**DOI:** 10.1186/s12871-019-0853-0

**Published:** 2019-10-24

**Authors:** Heba Omar, Wessam Adel Aboella, Mohammed Mahmoud Hassan, Amany Hassan, Passaint Hassan, Ahmed Elshall, Dalia Khaled, Maha Mostafa, Pierre Zarif Tawadros, Mona Hossam Eldin, Mai Wedad, Bassant Mohamed Abdelhamid

**Affiliations:** 1 Anesthesia Department, Faculty of Medicine, Cairo University, Cairo, Egypt; 2 El Sahel Teaching Hospital, Cairo, Egypt; 3 Anesthesia Department, National Cancer Institute, Cairo University, Cairo, Egypt

**Keywords:** Dexmedetomidine, Magnesium, Spinal, Shivering

## Abstract

**Background:**

Hypothermia and shivering are common complications after spinal anaesthesia, especially after uroscopic procedures in which large amounts of cold intraluminal irrigation fluids are used. Magnesium sulfate and dexmedetomidine are the most effective adjuvants with the least side effects. The aim of this study was to compare the effects of intrathecal dexmedetomidine versus intrathecal magnesium sulfate on the prevention of post-spinal anaesthesia shivering.

**Methods:**

This prospective randomized, double-blinded controlled study included 105 patients who were scheduled for uroscopic surgery at the Kasr El-Aini Hospital. The patients were randomly allocated into three groups. **Group C (*n*** **= 35) received** 2.5 ml of hyperbaric bupivacaine 0.5% (12.5 mg) + 0.5 ml of normal saline, **Group M (*n*** **= 35)** received 2.5 ml of hyperbaric bupivacaine 0.5% (12.5 mg) + 25 mg of magnesium sulfate in 0.5 ml saline, and **Group D (*n*** **= 35)** received 2.5 ml of hyperbaric bupivacaine 0.5% (12.5 mg) + 5 μg of dexmedetomidine in 0.5 ml saline. The primary outcomes were the incidence and intensity of shivering. The secondary outcomes were the incidence of hypothermia, sedation, the use of meperidine to control shivering and complications.

**Results:**

Group C had significantly higher proportions of patients who developed shivering (21), developed grade IV shivering (20) and required meperidine (21) to treat shivering than group M (8,5,5) and group D (5,3,6), which were comparable to each other.

The time between block administration and meperidine administration was similar among the three groups. Hypothermia did not occur in any of the patients.

The three groups were comparable regarding the occurrence of nausea, vomiting, bradycardia and hypotension. All the patients in group C, 32 patients in group M and 33 patients in group D had a sedation score of 2. Three patients in group M and 2 patients in group D had a sedation score of 3.

**Conclusions:**

Intrathecal injections of both dexmedetomidine and magnesium sulfate were effective in reducing the incidence of post-spinal anaesthesia shivering. Therefore, we encourage the use of magnesium sulfate, as it is more physiologically available, more readily available in most operating theatres and much less expensive than dexmedetomidine.

**Trial registration:**

Clinical trial registration ID: Pan African Clinical Trial Registry (PACTR) Trial Number PACTR201801003001727; January 2018, “retrospectively registered”.

## Background

For short procedures such as uroscopic surgeries, spinal anaesthesia (SA) is a very reliable and convenient technique, especially for procedures in which patient consciousness must be maintained to detect intraoperative complications, such as transurethral resection of the prostate (TURP) syndrome [1]. However, hypothermia and shivering are common complications after SA, especially when large amounts of cold intraluminal irrigating fluids are used [2]. SA impairs thermoregulation, inhibits tonic vasoconstriction, and causes the redistribution of core heat from the trunk to the peripheral tissue [3]. Shivering interferes with proper monitoring and is associated with several adverse effects, as it increases the circulating catecholamine, heart rate, cardiac output, minute ventilation, patient oxygen consumption, metabolic CO_2_ production, lactic acid level, intraocular and intracranial pressure, and postoperative pain from surgical incision stretching [4].

Various opioid and non-opioid agents, such as meperidine, ketamine, tramadol, and clonidine, have been used to prevent shivering, but they have many side effects, and their results have not been conclusive [5].

Dexmedetomidine is a highly selective alpha-2- adrenoreceptor agonist with potent effects on the central nervous system, decreasing the sympathetic tone [5]. It has been effectively used intravenously for the treatment [6–10] and prevention [11–14] of shivering following SA without any major adverse effects in several studies. Few trials have examined intrathecal dexmedetomidine for the prevention of post-SA shivering [5, 15].

Magnesium sulfate (MgSO_4_), which is an inorganic salt, has been shown to suppress postoperative shivering, indicating that the agent reduces the shivering threshold [16]. It has a good safety profile, as there are no side effects related to the intrathecal use of the drug and no significant changes in haemodynamic parameters have been reported [17]. Intravenous (IV) MgSO_4_ has been effectively examined in many trials [2, 18–20], but few trials involving intrathecal MgSO_4_ [3] to control shivering have been conducted.

The aim of this study was to evaluate and compare the effects of intrathecal dexmedetomidine versus intrathecal MgSO_4_ on the prevention of post-SA shivering.

The primary outcomes were the incidence and intensity of shivering. The secondary outcomes were the incidence of pethidine use, haemodynamic parameters and incidence of complications, including hypotension, bradycardia and sedation.

## Methods

After approval from the Ethics Committee of Cairo University Hospital (12015006), protocol registration in the Pan African Clinical Trial Registry (PACTR) (clinical trial registration ID: PACTR201801003001727, −retrospectively registered-) and obtaining informed written consent from each patient, this prospective randomized, double-blinded randomized controlled study was conducted at Kasr El-Aini Hospital in the urosurgical operating theatre following the Consolidated Standards for Reporting Trials (CONSORT) guidelines. A total of 105 patients scheduled for uroscopic surgery were enrolled. The **inclusion criteria** were as follows: patients aged between 20 and 60 years and patients with American Society of Anesthesiologists (ASA) class I or class II physical status. **The exclusion criteria** were as follows: patients who refused patients with coagulopathy, patients with a history of allergic reactions to local anaesthetics or patients with severe cardiac, respiratory, hepatic or renal diseases.

In the operating room**,** after lidocaine 2% was applied to the skin, venous puncture was performed with an 18 gauge cannula, and a preload of 500 ml of lactated Ringer’s solution was infused; no premedication was given. Five-lead electrocardiogram (ECG), pulse oximetry and non-invasive arterial blood pressure (NABP) monitoring was performed. The baseline systolic and diastolic arterial blood pressures (SBP and DBP), heart rate (HR) and arterial oxygen saturation (PSO_2_) were recorded. A spinal block was performed at the L3-L4 or L4-L5 interspace with a 22-gauge spinal needle after sterilization by povidone iodine while the patient was sitting and leaning forward; 2 cm of lidocaine 1% was applied to the skin at the site of lumbar puncture.

The patients were randomly allocated into three equal groups using computer-generated randomization tables. **Group C (*n*** **= 35) received** 2.5 ml of hyperbaric bupivacaine 0.5% (12.5 mg) + 0.5 ml of normal saline, **Group M (*n*** **= 35)** received 2.5 ml of hyperbaric bupivacaine 0.5% (12.5 mg) + 25 mg MgSO_4_ in 0.5 ml saline, and **Group D (*n*** **= 35)** received 2.5 ml of hyperbaric bupivacaine 0.5% (12.5 mg) + 5 μg dexmedetomidine in 0.5 ml saline. Patients’ assigned numbers and treatments were concealed in closed opaque envelopes that were retained by two members on the authors’ team who had no interaction with the patients. The specific intrathecal drug solutions were prepared and injected by an anaesthesiologist who was not involved in the study. The anaesthesiologists involved in patient observation and data collection were blinded to the treatment group, as were the patients.

The onset and durations of the motor and sensory blocks were assessed by the Bromage scale and pinprick test, respectively. The level of the block was assessed to ensure that it was between T10-T8; blocks higher than T8 and failed blocks were excluded. A surgical drape was placed over the patient, the room temperature was maintained at 24 °C, and all the irrigation and IV fluids were pre-warmed. No warming device was used. The incidence and intensity of shivering were assessed by a blinded observer immediately after the block was administered, every 5 min for the first 15 min, and then every 10 min for 2 h after the block using the Crossley and Mahajan scale [21] (0 = no shivering, 1 = piloerection or peripheral vasoconstriction but no visible shivering, 2 = muscular activity in only one muscle group, 3 = muscular activity in more than one muscle group but not generalized shivering, 4 = shivering involving the whole body). Twenty-five milligrams of IV meperidine was given when a patient presented grade 3 shivering. The core temperature was monitored using a tympanic probe before the block, immediately after the block and every 15 min for 2 h after the block. Hypothermia and active warming were considered if the core temperature reached 36 °C. The patient’s HR, BP and SPO_2_ were recorded every 5 min for the first 15 min and then every 10 min for 2 h after the block.

The sedation level was observed and recorded every 30 min for 2 h or until the administration of IV pethidine using the Ramsay sedation scale [22] (1 = the patient was anxious, agitated or restless; 2 = the patient was co-operative, oriented, and tranquil; 3 = the patient only responded to commands; 4 = the patient exhibited an immediate response to light glabellar tap or loud auditory stimulus; 5 = the patient exhibited a sluggish response to a light glabellar tap or loud auditory stimulus; and 6 = the patient exhibited no response).

The patients were monitored for complications. Hypotension (20% decrease in the SBP from baseline) was treated with incremental administration of 3 mg of ephedrine and 200 ml of lactated Ringer’s solution, and bradycardia (HR < 50) was treated with a bolus of 0.01–0.02 mg/kg of atropine. Nausea and vomiting were treated with 10 mg of metoclopramide.

Postoperatively, patients were transferred to the postanaesthesia care unit (PACU), monitored and covered with a cotton sheet. The PACU temperature was maintained at 25 °C.

The primary outcomes were the incidence and intensity of shivering. The secondary outcomes were incidence of hypothermia, sedation, the use of pethidine to control shivering and complications including hypotension, bradycardia, nausea and vomiting.

### Statistical analysis

#### Sample size

A power analysis using the chi-square test for independent samples was performed to analyse the frequency of patients complaining of intraoperative and early postoperative shivering because it was the main outcome variable in the present study. Previous studies showed that the frequency of patients with perioperative shivering was approximately 50% in patients who underwent elective minor lower abdominal operations under SA and 20% in patients who received dexmedetomidine [15]. Considering a power of 0.8 and an alpha error of 0.025, a minimum sample size of 35 patients was required for each group.

#### Method of analysis

The data were entered into the “Microsoft Office Excel” software program (2010) for Windows. The data were then entered into the Statistical Package for the Social Sciences software program, version 20 (IBM SPSS Statistics for Windows, Version 20.0. Armonk, NY: IBM Corp.), for statistical analysis.

The quantitative variables were presented as ranges and means plus standard deviations, and the qualitative variables were presented as frequencies and percentages.

Sex and ASA grade were presented as numbers and percentages. Age, height, weight, and sensory and motor block onset and duration were presented as means and standard deviations. Shivering incidence, ASA grades, the need for meperidine and complications were presented as numbers and percentages.

The Kolmogorov-Smirnov test was used to verify the normality of the distribution of the quantitative data described using means and standard deviations. Significance was considered at the 5% level. **Chi-square tests** were used to compare the categorical variables between the different groups. **F-tests (ANOVA)** were used to compare the normally distributed quantitative variables between the three groups, and post hoc tests (Tukey’s tests) were used for the pairwise comparisons.

## Results

**Fig. 1:**
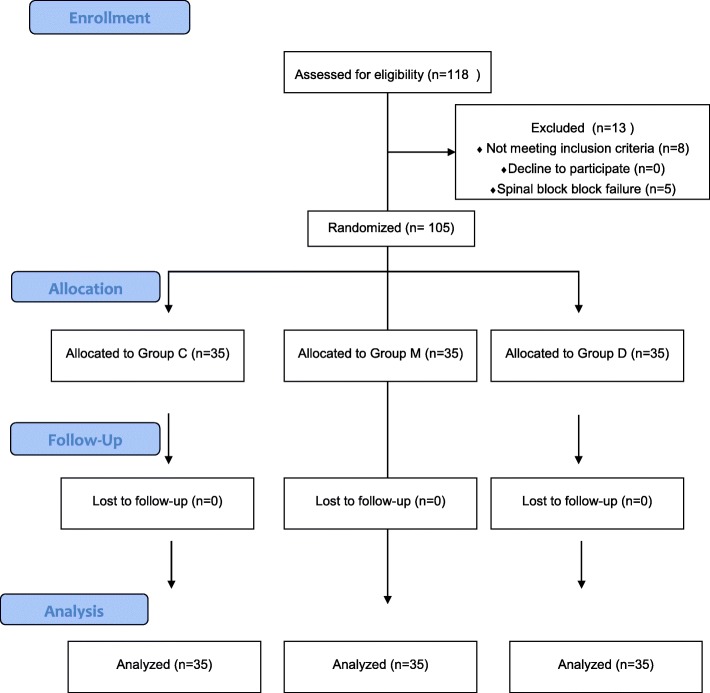
Consort flow chart

One hundred eighteen patients scheduled for uroscopic surgery were enrolled in this study; 8 patients did not meet the inclusion criteria, and 5 patients were excluded from the study due to failure to achieve spinal block within 15 min (Fig. 1). The types of the performed surgeries included cystoscopy (41 patients), TURP (17 patients), ureteroscopy (27 patients) and urethroscopy (20 patients); the proportions of patients according to the surgery types were comparable among the three groups.

All the patients were comparable regarding their demographic data, including age, weight, height, sex, duration of surgery and ASA classification (Table 1).

**Table 1:** Demographic data

	C Group (*n* = 35)	M Group (*n* = 35)	D Group (*n* = 35)	*P*	P1	P2	P3
Gender (No &%)							
Male	28 (80.0)	26 (74.3)	29 (82.9)	0.669	0.569	0.759	0.382
Female	7 (20)	9 (25.7)	6 (17.1)				
Age (years)	50.29 ± 8.87	49.03 ± 8.83	49.23 ± 10.51	0.836	0.579	0.640	0.930
Height (cm)	173.71 ± 5.60	174.29 ± 5.02	173.86 ± 5.83	0.903	0.644	0.914	0.745
Weight (kg)	83.43 ± 7.93	82.29 ± 9.34	82.71 ± 10.24	0.872	0.605	0.747	0.846
ASA classif1cation							
I	17 (48.6)	17 (48.6)	15 (42.9)	0.858	0.858	0.631	0.631
II	18 (51.4)	18 (51.4)	20 (57.1)				
Duration of surgery (min)	105.03 ± 11.59	103.17 ± 7.83	101.60 ± 9.01	0.331	0.420	0.138	0.495
Sensory Block onset (time to reach T10)	4.20 ± 0.63	6.69 ± 0.90	3.40 ± 0.50	< 0.001^*^	< 0.001^*^	< 0.001^*^	< 0.001^*^
motor block onset (time to reach bromage 4)	4.97 ± 0.71	8.03 ± 0.79	3.80 ± 0.76	< 0.001^*^	< 0.001^*^	< 0.001^*^	< 0.001^*^
Block level Median (IQR)	9 (8–10)	9 (8–10)	9 (8–10)	0.921	0.08	0.954	0.084
Motor block duration	157.00 ± 13.07	193.71 ± 17.63	206.57 ± 22.06	< 0.001^*^	< 0.001^*^	< 0.001^*^	0.003*
Sensory block duration	198.14 ± 18.67	243.43 ± 23.22	301.57 ± 39.44	< 0.001^*^	< 0.001^*^	< 0.001^*^	< 0.001^*^
Intraoperative venous fluids	971.43 ± 161.01	997.14 ± 168.45	1002.86 ± 263.45	0.652	0.448	0.503	.0.914
No of patients had cystoscopy (41)	15	13	13				
No of patients had uretroscopy (27)	8	10	9				
No of patients had urethroscopy (20)	7	6	7				
No of patients had TURP (17)	5	6	6				

p1: *p* value for comparing between Control and MgSO4

p2: *p* value for comparing between Control and Dex

p3: *p* value for comparing between MgSO4and Dex

*IQR* Interquartile range, *TURP* Transurethral resection of prostate

*: Statistically significant at *p* ≤ 0.05

**Fig. 2:**
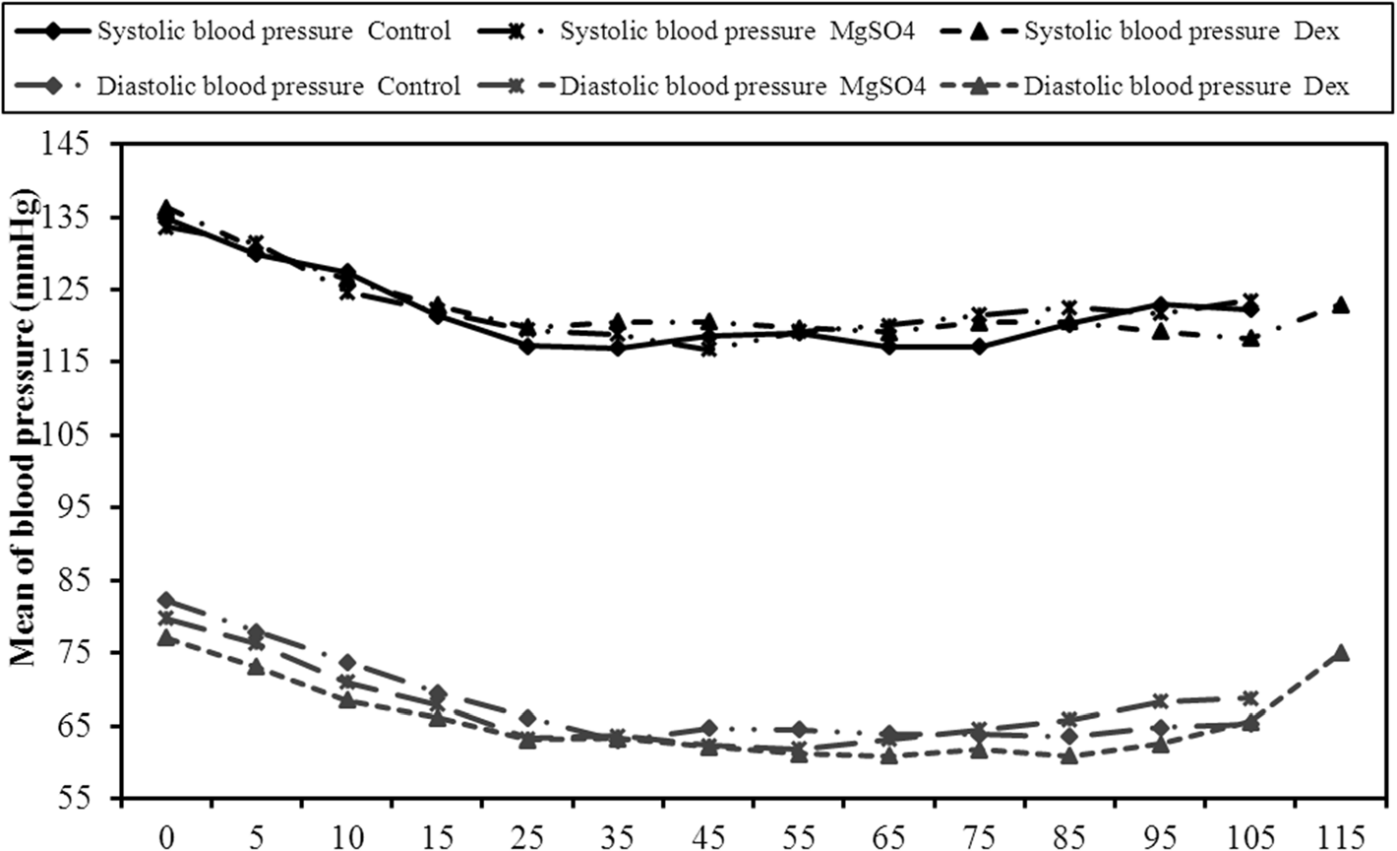
Comparison between the three studied groups according to Systolic blood pressure and diastolic blood pressure (mmHg)

**Fig. 3:**
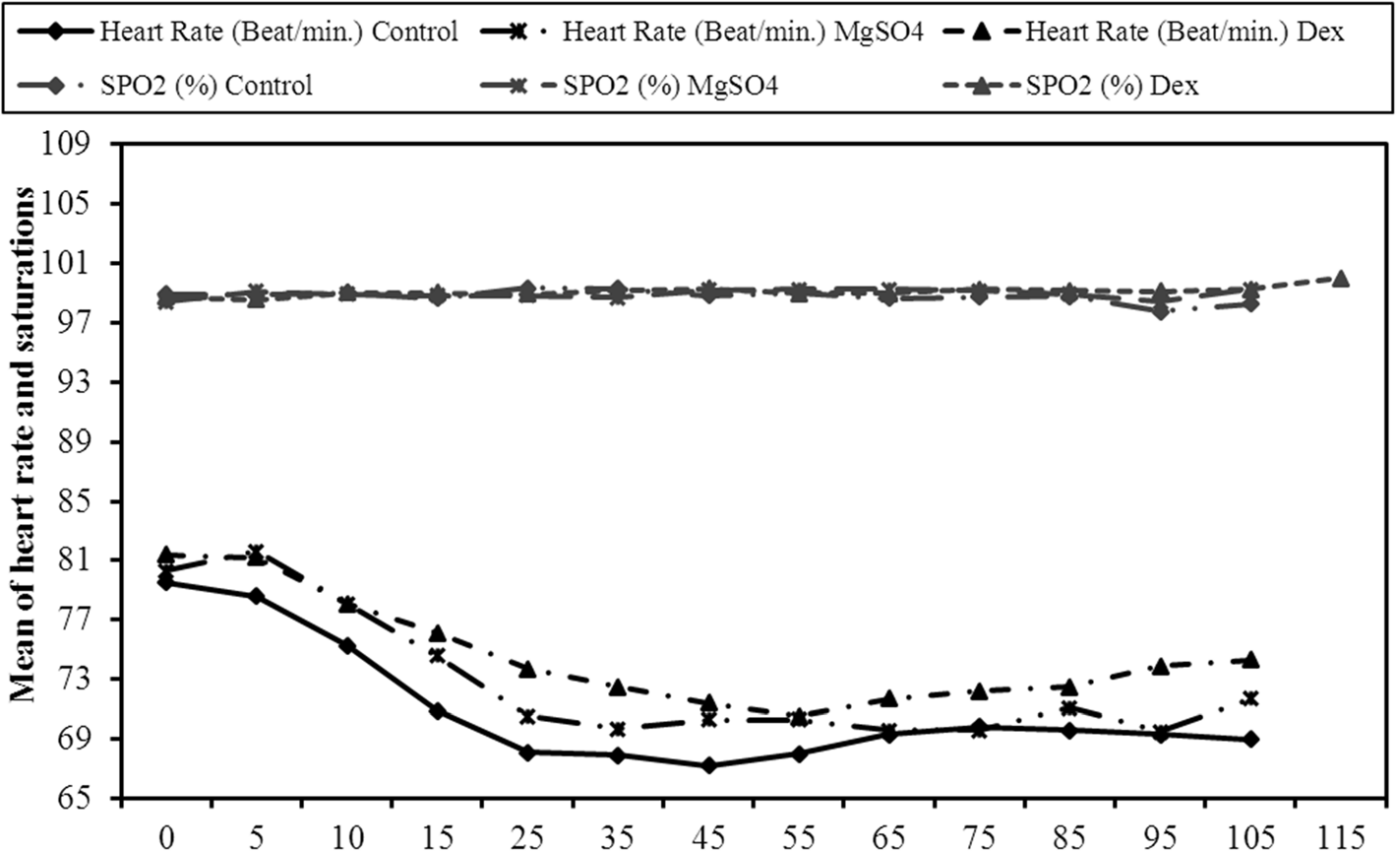
Comparison between the three studied groups according to Heart Rate (Beat/min.) and SPO_2_ (%)

All the patients were comparable regarding their SBP and DBP (Fig. 2), HR and SpO_2_ (Fig. 3) throughout the study.

The onset times and durations of the sensory and motor blocks were statistically different between the three groups. Group D presented the shortest onset, and group M presented the longest onset (*p* < 0.001). All the patients were comparable regarding their block levels (Table 1).

**Fig. 4:**
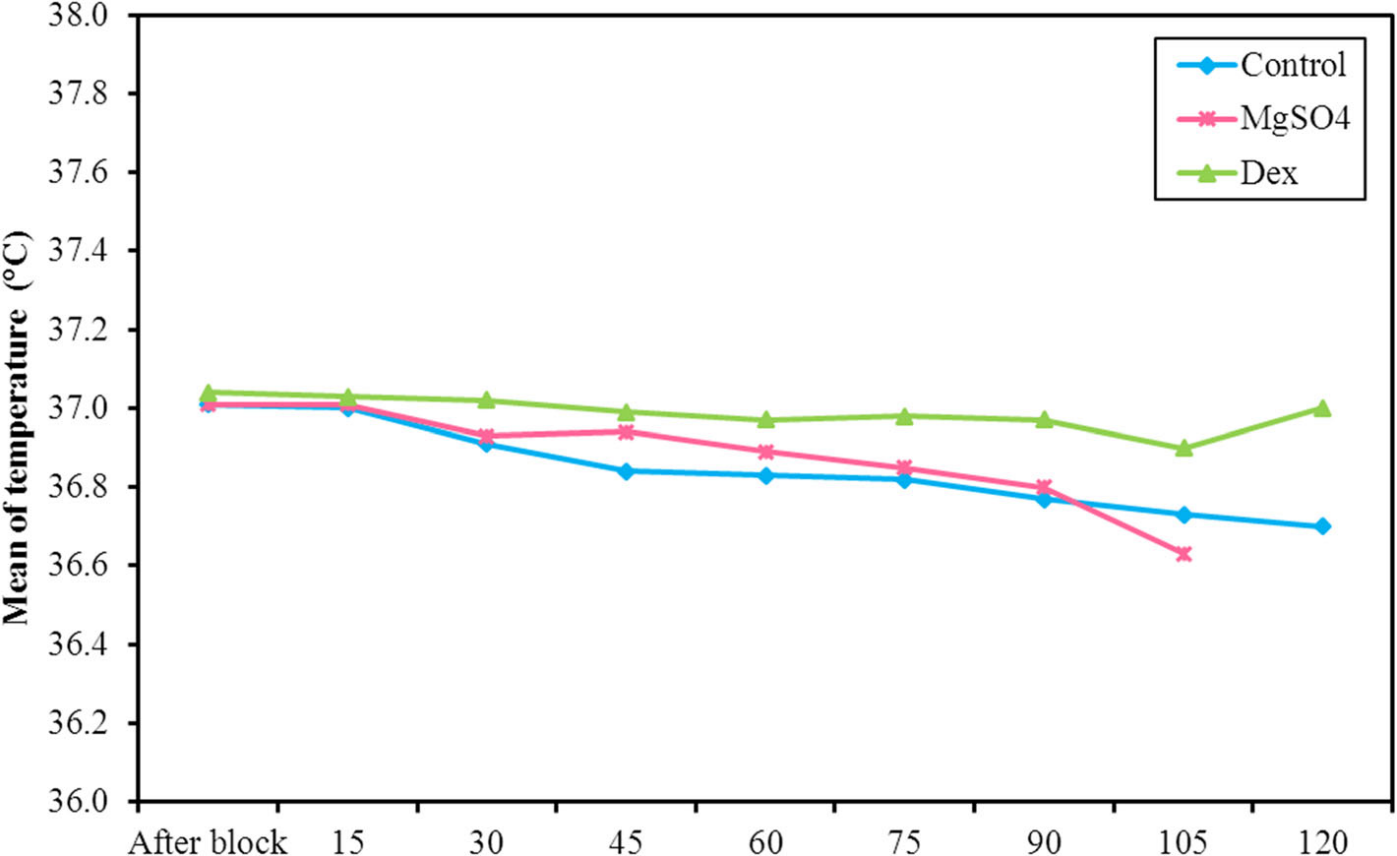
Comparison between the three studied groups according to temperature (°C)

The tympanic temperature remained above 36 °C in all the patients at all times, and no patient required active warming (Fig. 4).

Group C had a significantly higher number of patients who developed shivering, who developed grade IV shivering and who needed meperidine to treat shivering than groups M and D, which were comparable to each other (Tables 2 and 3).

**Table 2:** Shivering incidence and grades

	C Group (*n* = 35)		M Group (*n* = 35)		D Group (*n* = 35)		*P*	p_1_	p_2_	p_3_
	No.	%	No.	%	No.	%				
Grades										
No shivering	14	40.0	24	68.6	30	85.7	< 0.001^*^	0.016^*^	< 0.001^*^	0.088
I	0	0.0	0	0.0	0	0.0	–	–	–	–
II	0	0.0	2	5.7	0	0.0	0.331	0.493	–	0.493
III	1	2.9	1	2.9	2	5.7	1.000	1.000	1.000	1.000
IV	20	57.1	5	14.3	3	8.6	< 0.001^*^	< 0.001^*^	< 0.001^*^	0.716

p1: *p* value for comparing between Control and MgSO4

p2: *p* value for comparing between Control and Dex

p3: *p* value for comparing between MgSO4and Dex

*: Statistically significant at *p* ≤ 0.05

**Table 3:** Need of meperdine

	C Groupo		M Group		D Group		*P*	p_1_	p_2_	p_3_
	No.	%	No.	%	No.	%				
No. of patients needed meperdine	21	60.0	6	17.4	5	14.3	< 0.001^*^	< 0.001^*^	< 0.001^*^	0.743
Timing after block/min.	39.52 ± 8.05		44.50 ± 11.17		52.0 ± 14.40		0.175	0.586	0.058	0.268
Recurrence of shivering	6	17.1	2	5.7	1	2.8	0.136	< 0.001^*^	< 0.001^*^	0.002^*^

p1: *p* value for comparing between Control and MgSO4

p2: *p* value for comparing between Control and Dex

p3: *p* value for comparing between MgSO4and Dex

*: Statistically significant at *p* ≤ 0.05

The time between block administration and meperidine administration was similar among the three groups.

The three groups were comparable regarding the occurrence of nausea, vomiting, bradycardia and hypotension. All the patients in group C, 32 patients in group M and 33 patients in group D had a sedation score of 2. Three patients in group M and 2 patients in group D had a sedation score of 3 (Table 4).

**Table 4:** Complications

Complication	C Group (*n* = 35)		M Group (*n* = 35)		D Group (*n* = 35)		*P*	p_1_	p_2_	p_3_
	No.	%	No.	%	No.	%				
Nausea	3	8.6	2	5.7	2	5.7	1.000	1.000	1.000	1.000
Vomiting	0	0.0	0	0.0	0	0.0	–	–	–	–
Bradycardia	4	11.4	4	11.4	3	8.6	1.000	1.000	1.000	1.000
Hypotension	7	20.0	6	17.1	7	20.0	0.940	0.759	1.000	0.759
Sedation (maximum score)										
1	0	0.0	0	0.0	0	0.0	0.364	0.239	0.493	1.000
2	35	100.0	32	91.4	33	94.3				
3	0	0.0	3	8.6	2	5.7				
4	0	0.0	0	0.0	0	0.0				
5	0	0.0	0	0.0	0	0.0				
6	0	0.0	0	0.0	0	0.0				

p1: *p* value for comparing between Control and MgSO4

p2: *p* value for comparing between Control and Dex

p3: *p* value for comparing between MgSO4and Dex

## Discussion

In addition to its well-known advantages, SA has an additional advantage in uroscopic surgeries, especially procedures that require intraluminal fluid irrigation such as TURP, as it allows the early detection of complications such as TURP syndrome in conscious patients [1]. However, SA is not a complication-free technique; shivering is a common complication of SA, with an incidence of 40–60% in patients who undergo SA [5]. Though shivering is a protective mechanism to preserve body heat, it causes patient discomfort and pain and may be dangerous in patients with impaired cardiovascular reserves or limited respiratory capacity, as shivering increases the circulating catecholamine, HR, cardiac output, minute ventilation, oxygen consumption, metabolic CO_2_ production and lactic acid level. It also increases intraocular and intracranial pressure and postoperative pain due to surgical incision stretching. Shivering may also interfere with the monitoring of patients by causing artefacts on the ECG or disrupting BP and pulse oximetry readings [4]. Additionally, shivering in patients with ASA grades III and IV may cause challenges for the surgeon and increase the operative time.

Hypothermia is a major risk for shivering, but there is no definite linear relationship between body temperature and the occurrence of shivering. Other major risk factors include age, sensory block level, temperature of the operating room and temperatures of the IV solutions [23].

The exact mechanisms to explain the occurrence of shivering during SA have not yet been elucidated. Possible mechanisms include central thermoregulation disturbance, internal body heat redistribution, and body heat loss to the environment [24]. Regional and general anaesthesia are known to impair the efficiency of the hypothalamic thermoregulatory centre, causing different grades of hypothermia [25]. Under regional anaesthesia, vasodilatation and redistribution of the core temperature are restricted to the lower body below the block, while vasoconstriction and shivering are restricted to the upper body, as they are inhibited below the level of the block due to sympathetic and somatic nerve blocks [26].

The neurotransmitter pathways responsible for shivering are complex, and different receptors patients, such as opioid, α-2 adrenergic, serotonergic, and anti-cholinergic receptors, are involved patients. Different drugs that act on these receptors have been examined in different trials for the prevention or treatment of shivering after SA [4]. The studied drugs include meperidine, fentanyl, clonidine, ketamine, and tramadol; they have resulted in different degrees of efficacy and many associated side effects, such as haemodynamic instability, respiratory depression, nausea and vomiting [27].

Dexmedetomidine [5–15] and magnesium sulfate [2, 3, 17–20] have been demonstrated to be effective and safe for the prevention and treatment of shivering following SA; their efficacy and safety are equal or superior to other adjuvants, with fewer adverse effects. Few trials [3, 5, 15, 17] have examined intrathecal administration to prevent SA-related shivering.

Dexmedetomidine is a highly selective alpha-2 adrenergic agonist, known for its sedative effect in anaesthesia and intensive care practice; its affinity for alpha-2 adrenoreceptors is ten times higher than that of clonidine [28]. The response to activation of these alpha-2- receptors includes a decreased sympathetic tone, resulting in a decreased BP and HR. Dexmedetomidine has been effectively examined in several studies for the prevention and treatment of shivering following general anaesthesia or SA at a dose that does not induce major sedation or haemodynamic instability, respiratory depression, nausea or vomiting [11]. The exact mechanism of dexmedetomidine in the control of shivering is unclear and complex. One suggested mechanism is as follows: dexmedetomidine and other alpha-2 agonists reduce shivering by inhibiting central thermoregulatory control by restraining neuronal conductance and suppressing vasoconstriction and shivering thresholds. Drugs that inhibit thermoregulatory vasoconstriction prevent shivering [29].

Magnesium (Mg^+ 2^) is a naturally occurring non-competitive antagonist of N-methyl-D aspartate (NMDA) receptors with a good safety profile and neuroprotective properties under the condition of hypothermia [30]. There are many theories regarding the anti-shivering effect of MgSO_4_; it has been reported to reduce shivering through a central effect by reducing the shivering threshold [16, 31]. Additionally, it blocks NMDA receptors and decreases norepinephrine and 5-HT, which both play a role in thermoregulation [32]. As a calcium antagonist, Mg^+ 2^ has a peripheral mild muscle relaxation effect that may reduce the intensity of shivering (incremental shivering intensity with progressing hypothermia) [33]. Mg^+ 2^ also cause peripheral vasodilation, which increases cutaneous circulation, leading to a decrease in the incidence of shivering [34, 35].

Based on previous studies, we compared the effect of subarachnoid injections of dexmedetomidine and MgSO_4_ on the prevention of shivering after SA in patients who underwent uroscopic surgery. The current study demonstrated that post-SA injection of both dexmedetomidine (5 μg) and MgSO_4_ (25 mg) significantly reduced the incidence of post-SA shivering. Five patients (14.3%) in group D, 8 patients (22.8%) in group M, and 21 patients (60%) in group C developed shivering. The number of patients who developed shivering and required meperidine administration in group D group was 5 (14.3%), in group M was 6 (17.4%), and in group C was 21 (60.0%).

Similar to our study, Ellakany et al. [15] administered the same intrathecal dexmedetomidine dose (5 μg) and concluded that both intrathecal dexmedetomidine and meperidine effectively lowered the incidence of shivering following SA in patients who underwent lower abdominal surgery, but meperidine was associated with more side effects, such as pruritus, nausea and vomiting, than dexmedetomidine. In sixty patients who underwent lower abdominal surgery, Abdel Hamid et al. [36] concluded that adding 5 μg of dexmedetomidine to intrathecal bupivacaine improved the characteristics of the spinal block, with less postoperative analgesic requirements and a lower incidence of shivering than the placebo with no sedation or other complications.

Moawad et al. [5] studied the effect of adding dexmedetomidine to intrathecal bupivacaine at a higher dose (10 μg) than that in our study (5 μg). They found that it significantly decreased the incidence and degree of shivering in patients undergoing TURP.

Usta et al. [12] and Bajwa et al. [23] studied the prophylactic effect of IV dexmedetomidine on shivering in patients who received SA and general anaesthesia, respectively. They found that perioperative dexmedetomidine infusion significantly decreased the incidence and intensity of shivering, with no major adverse effects.

Botros et al. [14] compared the prophylactic effects of an intravenously infused placebo, 1 μg/kg of dexmedetomidine and 8 mg of ondansetron on the prevention of post-SA shivering in 120 patients undergoing different lower body surgeries and found that IV dexmedetomidine and ondansetron were equally as effective in reducing the incidence of post-SA shivering as the placebo.

Abdel-Ghaffar et al. [6] compared the clinical efficacy and side effects of three different doses of IV dexmedetomidine (0.5, 0.3 and 0.2 μg/kg) and IV meperidine 0.4 mg/kg for the treatment of post-SA shivering in 120 patients. They found that 0.3 μg/kg of dexmedetomidine was the most appropriate dose for the effective treatment of shivering after SA, with modest effects on haemodynamic characteristics and sedation.

Faiz et al. [3] concluded that an intrathecal injection of MgSO_4_ (25 mg) improved perioperative shivering in female patients who underwent an elective caesarean section.

Ibrahim et al. [19] studied the prophylactic and therapeutic effects of IV MgSO_4_ on SA-induced shivering and proved that it was effective.

Gozdemir et al. [2] found that following SA, an IV infusion of 80 kg/mg of MgSO_4_ over 30 min, followed by IV infusion at a rate of 2 g/hr. until the end of surgery had a significant effect on preventing post-SA shivering in patients undergoing TURP.

In a study by Sachidananda et al. [20], a prophylactic IV infusion of MgSO_4_ and tramadol effectively reduced shivering during caesarean section under SA and the shivering intensity.

There was no difference between the three groups in terms of intraoperative haemodynamics. Regarding the sensory and motor block onset times, group D had the fastest sensory and motor blocks onset times, while group M had delayed sensory and motor block onset times compared to group D. This may be due to changes in the pH and baricity of bupivacaine due to the addition of MgSO_4_. The durations of both the sensory and motor blocks in group D were longer than those in group M, which were longer than those in group C. Similar results were observed in two studies [37, 38] that compared the effects of 10 μg of intrathecal dexmedetomidine and 50 mg of intrathecal MgSO_4_ when added to bupivacaine, considering the characteristics of the spinal block as the primary outcomes.

## Limitations and recommendations

The limitation to this study was that we did not estimate the mean volumes of the irrigating fluids in each group. We recommend conducting further studies on both drugs with increased sample sizes and different doses.

## Conclusion

We concluded that intrathecal injection of both dexmedetomidine and MgSO_4_ with bupivacaine were effective in reducing the incidence of post-SA shivering. Therefore, we encourage the use of MgSO_4_,as is it more physiologically available, more readily available in most operating theatres and much less expensive than dexmedetomidine.

## Data Availability

The data that support the findings of this study are the possession of the Cairo University Hospital. However, data are available from the corresponding author upon reasonable request after permission from Cairo University.

## Abbreviations

ASA: American Society of Anesthesiologists
°C: Degrees Celsius
CO_2_: Carbon dioxide
DBP: Diastolic arterial blood pressure
ECG: Electrocardiogram
HR: Heart rate
IV: Intravenous
Mg^+ 2^: Magnesium
MgSO_4_: Magnesium sulfate
NABP: Non-invasive arterial blood pressure
NMDA: N-methyl-D-aspartate
PACU: Postanaesthesia care unit
PSO_2_: Arterial oxygen saturation
SA: Spinal anaesthesia
SBP: Systolic arterial blood pressure
TURP: Transurethral resection of the prostate
μg: Microgram
